# Neural illumination calibration for surgical workflow-optimized spectral imaging

**DOI:** 10.1007/s11548-025-03525-8

**Published:** 2025-10-07

**Authors:** Alexander Baumann, Leonardo Ayala, Alexander Studier-Fischer, Jan Sellner, Berkin Özdemir, Karl-Friedrich Kowalewski, Slobodan Ilic, Silvia Seidlitz, Lena Maier-Hein

**Affiliations:** 1 Siemens AG, Munich, Germany; 2 Division of Intelligent Medical Systems, German Cancer Research Center (DKFZ) Heidelberg, Heidelberg, Germany; 3 Helmholtz Information and Data Science School for Health, Karlsruhe/Heidelberg, Germany; 4 Faculty of Mathematics and Computer Science, Heidelberg University, Heidelberg, Germany; 5 National Center for Tumor Diseases (NCT), NCT Heidelberg, a partnership between DKFZ and university medical center Heidelberg, Heidelberg, Germany; 6 Department of General, Visceral, and Transplantation Surgery, Heidelberg University Hospital, Heidelberg, Germany; 7 Medical Faculty, Heidelberg University, Heidelberg, Germany; 8 Department of Urology and Urosurgery, University Medical Center Mannheim, Mannheim, Germany; 9 Division of Intelligent Systems and Robotics in Urology (ISRU), German Cancer Research Center (DKFZ) Heidelberg, Heidelberg, Germany; 10 DKFZ Hector Cancer Institute, University Medical Center Mannheim, Mannheim, Germany

**Keywords:** Illumination Calibration, Hyperspectral Imaging, Intra-Operative Imaging, Deep Learning

## Abstract

**Purpose:**

Hyperspectral imaging (HSI) is emerging as a promising novel imaging modality with various potential surgical applications. Currently available cameras, however, suffer from poor integration into the clinical workflow because they require the lights to be switched off or the camera to be manually recalibrated as soon as lighting conditions change.

**Methods:**

We propose a novel learning-based approach to recalibration of hyperspectral cameras during surgery that predicts the corresponding white reference image from an uncalibrated hyperspectral input, enabling spatially resolved, automatic, and sterile calibration under varying illumination conditions. Our key novelty lies in (i) the disentanglement of the space of possible illuminations from the space of possible tissue configurations and (ii) combining real-world white reference measurements with physics-inspired simulated illuminations to create a diverse and representative training set.

**Results:**

Based on a total of 1,890 HSI cubes from a phantom, porcine subjects, rats, and humans, we derive the following key insights: Firstly, dynamically changing lighting conditions in the operating room dramatically reduce the performance of methods for physiological parameter estimation and surgical scene segmentation. Secondly, our method is not only sufficiently accurate to replace the tedious process of white reference-based recalibration, but also outperforms previously proposed methods by a large margin. Finally, our approach generalizes across species, lighting conditions, and image processing tasks.

**Conclusion:**

Our method enables seamless integration of hyperspectral imaging into surgical workflows by providing rapid and automated illumination calibration. Its robust generalization across diverse conditions significantly enhances the reliability and practicality of spectral imaging in clinical settings, paving the way for broader adoption of HSI in surgery.

**Supplementary Information:**

The online version contains supplementary material available at 10.1007/s11548-025-03525-8.

## Introduction

**Fig. 1:**
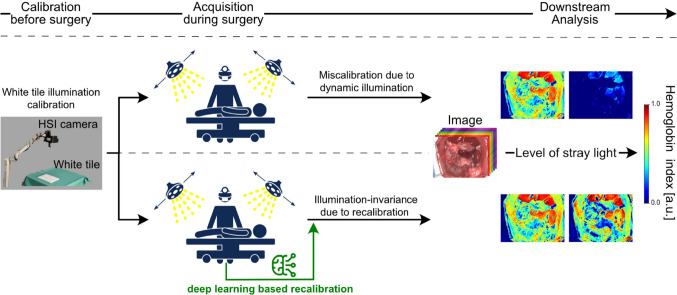
Current hyperspectral cameras, reliant on static illumination environments, fail in real-world scenarios with dynamically changing lighting conditions

Hyperspectral imaging (HSI) has gained prominence in medical imaging, offering enhanced spectral information over conventional RGB imaging. Recent studies highlight its benefits in tissue classification [1–5] and its potential for estimating physiological tissue parameters [6–12]. However, in open surgery, spectral data are susceptible to variations in illumination, necessitating proper calibration whenever lighting conditions change [13]. Current experimental protocols require switching off all external light sources for accurate data capture [14]. Given that these measures considerably disrupt the clinical workflow, it is presumed not to be consistently applied, leading to compromised data integrity and degraded downstream task performance, as illustrated in Fig. 1. This may contribute to the limited clinical adoption of spectral imaging.

Conventional HSI light calibration utilizes a physical white tile measurement, representing the surrounding illumination. This method, however, is constrained by temporal and sterility factors, rendering it impractical in the operating room (OR) context. While the proposed use of white sterile OR rulers [15] addresses sterility concerns, it introduces challenges due to inaccuracies and an increased workload. Several automatic calibration algorithms, originally developed for RGB imaging, can be adapted for HSI, recovering a global scene illuminant based on intensity statistics [16–18]. Alternatively, HSI-specific calibration leverages specular highlights for illuminant estimation using similar statistical methods [19]. However, learning-based methods have demonstrated superior performance over their non-learning counterparts for RGB calibration [20, 21]. Recently a deep learning approach tailored for multispectral illumination calibration was developed [22]. Adapting an RGB-based technique [20], this method processes multispectral channel triplets in the log-chroma space using convolutional operations. However, applying this approach to hyperspectral images results in high computational costs due to the exponential increase in channel triplet combinations. More critically, all preceding methods assume spatially uniform illumination—an unrealistic simplification for OR environments [23]. In RGB imaging, multi-illuminant color constancy models have shown promise in overcoming this challenge, predicting pixel-wise illuminants for calibration [24–26]. In spectral imaging, however, only one deep learning approach has thus far addressed multi-illuminant calibration [27]. Specifically, Li et al. framed illumination calibration as a factorization problem between surface reflectance and illumination intensity, proposing an optimization framework based on unrolling networks to extract the surface reflectance information.

Overall, the methods proposed in the literature either remain untested for surgical HSI, which presents illumination characteristics distinct from natural scenes [23], and/or are conceptually not suitable for spatially resolved calibration. Given this bottleneck, the mission of our work was to develop a new workflow-optimized calibration approach that enables widespread clinical spectral imaging. Our specific contribution is fourfold:

1. *Limitations of current methods:* We demonstrate that dynamically changing lighting conditions in the OR dramatically affect the performance of in vivo HSI applications, and previously proposed calibration methods fail to restore optimal performance.
2. *Need for spatially resolved calibration:* We show that spatially uniform illuminants cannot adequately represent the illumination within the OR, underlining the necessity of spatially resolved illumination calibration methods.
3. *New learning-based approach:* We present a novel learning-based approach to performing spatially resolved light recalibration of surgical hyperspectral images. Specifically, we propose to replace conventional physical white reference measurements with a data-driven prediction of the corresponding white tile measurement. This enables a seamless and sterile recalibration process during surgery.
4. *Large-scale validation across three species:* Based on the downstream tasks of semantic segmentation and physiological parameter estimation, we show that our recalibration method not only outperforms previous methods, but also generalizes across species, lighting conditions, and image processing tasks.

With this contribution, we substantially expand our preliminary version published at the International Conference on Medical Image Computing and Computer Assisted Intervention (MICCAI) [28] by (i) validating the benefit of our method on a human patient cohort with a total of 1,148 hyperspectral images, (ii) investigating the additional research question on spatially resolved illuminants, (iii) performing comprehensive and fine-grained analyses of core design choices, and (iv) including two additional baseline methods.

## Materials and methods

**Fig. 2:**
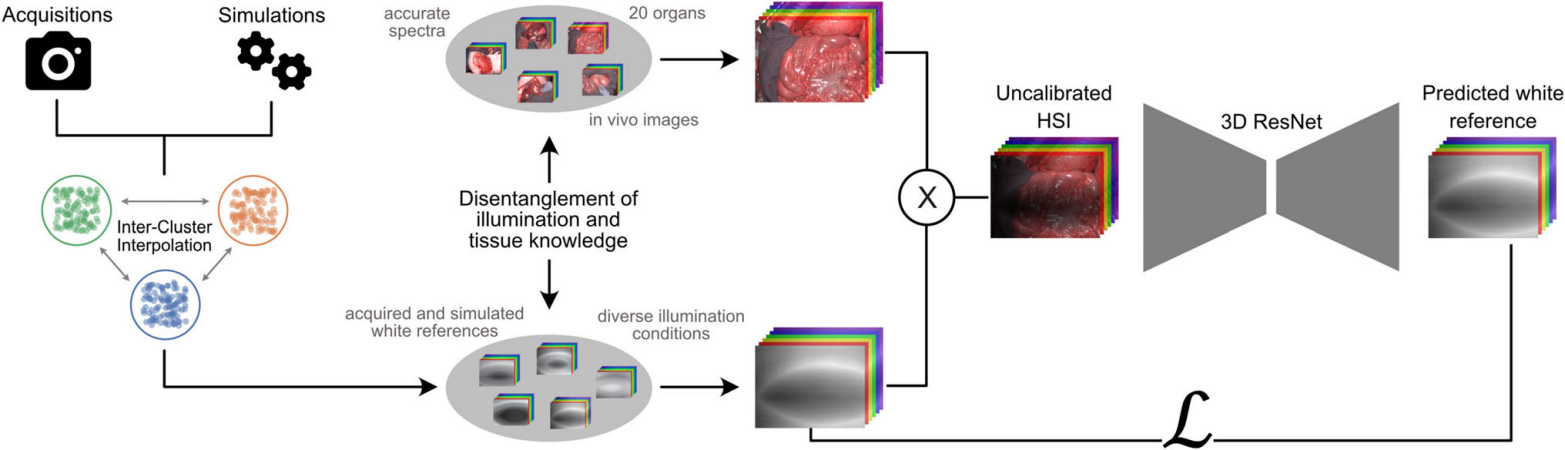
The proposed approach substitutes labor-intensive manual calibration with a dynamic fully-automated approach. At the heart of our data-centric method lies a 3D convolutional neural network, trained on in vivo data augmented with synthetic illumination variations. At inference, the model processes a raw hyperspectral image and generates the corresponding white reference image. The resulting white tile prediction facilitates the subsequent calibration of the input image

The primary challenge in data-driven calibration lies in generalizing to unseen settings To render our method conceptually robust to domain shifts, we propose estimating the white tile measurement corresponding to a given scene rather than directly predicting the recalibrated image. The underlying premise of our methodology is that the acquisition of a comprehensive training dataset, encompassing all potential tissue and illumination configurations, is practically unattainable. We therefore disentangle the space of possible illuminations from the space of possible tissue configurations, as illustrated in Fig. 2. Specifically, a two-dataset training paradigm is implemented for the neural network. The first dataset, referred to as the illumination dataset, comprises a collection of real and simulated white tile images, capturing diverse lighting conditions prevalent within the OR. The second dataset consists of accurately calibrated images of clinically relevant samples. To simulate a stray light-affected HSI cube, an image in the sample dataset is augmented through element-wise multiplication with a white reference image. Subsequently, the neural network is trained to reconstruct the illumination from the input. During inference, an uncalibrated hyperspectral image, possibly contaminated by stray light, is fed into the neural network to predict the white reference image required for illumination calibration.

To contextualize the design choices of our model, we first provide a concise theoretical overview of how light influences image formation, based on [29], before elaborating on our illumination generation strategy and neural network architecture.

### Theoretical background

Assuming negligible specular reflections, image formation can be modeled as integral of the product of surface reflectance *R*, illumination intensity *L*, and the camera’s spectral sensitivity *S*, which determines the captured wavelength range for each channel. Specifically, an image *I* is modeled as:

1 $$\begin{aligned} I_c(x,y) = \int _{\Lambda _c} R(x,y,\lambda )L(x,y,\lambda )S_c(\lambda )d\lambda \end{aligned}$$

where (*x*, *y*) denotes the spatial coordinates, *c* the channel index, and $\Lambda _c = \{\lambda \in \mathbb {R} | S_c(\lambda ) \ne 0\}$ represents the support of $S_c$. Consequently, a white reference image, $I_{\text {white}}$, representing the surrounding illumination, enables calibration through element-wise division, as expressed by $I_{\text {cal}}=I \oslash I_{\text {white}}$. Synthetic relighting of a calibrated scene is then accomplished by element-wise multiplication with an arbitrary illuminant.

### Datasets

**Fig. 3:**
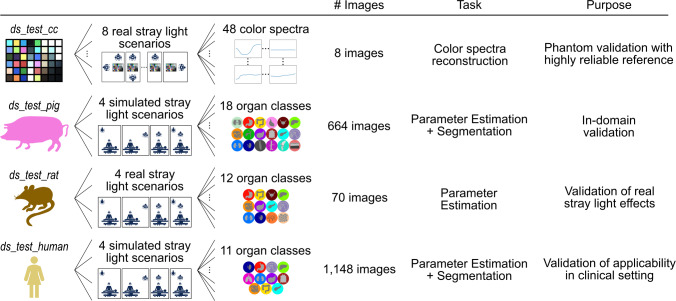
Testing concept based on data from a phantom and three species

Model training and validation were performed exclusively on porcine data, whereas testing systematically assessed stray light conditions on unseen porcine subjects, a phantom, rats, and humans, as summarized in Fig. 3. While the phantom colorchecker board dataset was acquired with the Tivita^®^ 2.0 Surgery (Diaspective Vision GmbH, Am Salzhaff, Germany) featuring light-emitting diode (LED) illumination, the others were captured with the halogen-based Tivita^®^ Tissue. As these light sources exhibit different behavior when interfering with the main stray light source, namely LED-based surgical lights [23], we evaluated on both systems.

*Measured illumination dataset:* The purpose of this dataset was to acquire a variety of representative OR lighting conditions for algorithm training. To this end, white reference images were acquired within an OR, accounting for camera light and various stray light sources, including Dr. Mach LED surgical lights (Model: 8MC), ceiling lights, and daylight. Diverse stray light scenarios were achieved by varying the angle, distance, and number of surgical lights as well as adjusting blinds or ceiling light, resulting in a wide range of illumination spectra. As the two HSI systems in this study differ in the light sources, we captured one illumination dataset with each camera: $ds\_dev\_ill\_led$ (LED) and $ds\_dev\_ill\_hal$ (halogen).

*In vivo porcine development dataset:* For model development, we curated the publicly available HSI dataset HeiPorSPECTRAL [14], partitioning it into a training set, $ds\_dev\_pig$, and a validation set, $ds\_val\_pig$. Notably, these datasets exhibit no overlap with the downstream datasets used for testing.

*Test datasets:* The proposed methodology underwent thorough evaluation utilizing the four datasets $ds\_test\_cc$, $ds\_test\_pig$, $ds\_test\_rat$, and $ds\_test\_human$—detailed in Fig. 3. To evaluate the model’s calibration efficacy, the evaluation strategy comprised both simulated and real stray light scenarios, as recommended by [29–31]. Colorchecker boards acquired under diverse lighting conditions assessed recalibration performance based on high-fidelity reference data ($ds\_test\_cc$). In-domain testing was performed on $ds\_test\_pig$, while the model’s generalizability and clinical applicability were evaluated on $ds\_test\_rat$ and $ds\_test\_human$.

Both $ds\_test\_pig$ and $ds\_test\_human$ were acquired under controlled, constant lighting conditions to ensure accurate calibration; additional details can be found in [1, 32]. To simulate stray light effects in these datasets, we generated an illumination dataset, $ds\_test\_ill\_hal$, comprising white reference images captured under four distinct lighting setups. These setups vary in both the intensity and position of the stray light source: (1) The first scenario uses only ceiling light as stray light, (2) the second consists of a Dr. Mach LED surgical light illuminating the surgical field from the right, (3) the third positions the surgical light to shine directly onto the surgical site, and (4) the fourth combines both ceiling light and direct surgical light illumination on the site.

In contrast, the rat organ images were acquired under real stray light conditions. Each scene was first captured without stray light and subsequently re-imaged under the four illumination setups described above. For each illumination configuration, two acquisitions were made, resulting in a total of 10 images per subject. The complete process for acquiring all lighting setups per subject took approximately 4.5 min, during which we ensured the rat’s physiological stability and maintained a consistent scene.

### Physics-based illumination simulation

To implement the data-centric recalibration concept, we focused on the model-based generation of plausible white tile data. To overcome the resource-intensive acquisitions, we enhanced both $ds\_dev\_ill\_led$ and $ds\_dev\_ill\_hal$ by synthesizing illumination images from real white tile images. Since surgical overhead lights, predominantly LED-based, are the main source of stray light in ORs [23] and induce distinct interference patterns in our LED and halogen-based HSI systems, we developed tailored simulation strategies for each. While LED-based simulations comprise interpolations of entire hyperspectral images, our halogen-based strategy first simulates one-dimensional light spectra and subsequently synthesizes full hyperspectral images by incorporating realistic spatial variations in intensity.

*LED simulations:* For the LED-based HSI system, wave interference with LED-based surgical lights is approximately constructive; thus, local extrema in the spectrum of the camera light source are preserved. To model this interference behavior, we employed an efficient interpolation strategy. Initially, we grouped existing white tile images into $K=4$ clusters through the K-means algorithm, with each cluster representing a distinct illumination scenario. This clustering was performed on spatially averaged spectra, providing sparse representations of the illuminations. To create new white tile images, we then conducted inter-cluster interpolation by combining randomly selected images from different clusters.

*Halogen simulations:* Halogen-based HSI systems, unlike their LED-based counterparts, experience destructive wave interference when used alongside LED surgical lights due to differences in local extrema. Consequently, standard interpolation techniques, which inherently preserve local extrema, prove inadequate. To address this limitation, we introduce a simulation strategy that can shift extrema positions, effectively accommodating this spectral variability. The complementary roles of interpolations and simulations are illustrated in Suppl. Figure 1. Our simulation approach synthesizes hyperspectral images by first modeling one-dimensional light spectra using a parametric function and subsequently including realistic spatial variations in intensity. Inspired by Planck’s radiation law and empirical observations, we define the parametric function as follows:

2 $$\begin{aligned} f_{p_1,p_2,p_3,p_4}(\lambda ) = \frac{(p_1\lambda - p_2)^3}{\exp (p_3\lambda - p_4) - 1} \end{aligned}$$

where $f_{p_1,p_2,p_3,p_4}$ is the intensity, $\lambda$ is the wavelength and $p_1,\dots ,p_4$ are the parameters. For each instance of $ds\_dev\_ill\_hal$, least-squares optimization was conducted to determine parameters that best approximate the given instance. Subsequently, the mean $\mu _k$ and standard deviation $\sigma _k$ of parameter $p_k$ were calculated across all instances. Using a fixed number of standard deviations, $n_\sigma$, intervals $P_k$ for parameter $p_k$ were defined as $P_k=[\mu _k - n_\sigma \sigma _k; \mu _k + n_\sigma \sigma _k]$. Subsequently, sampling $(\tilde{p}_1,\tilde{p}_2,\tilde{p}_3,\tilde{p}_4)$ from the grid $P_1\times P_2\times P_3\times P_4$ yields a simulated light spectrum $f_{\tilde{p}_1,\tilde{p}_2,\tilde{p}_3,\tilde{p}_4}$. Illumination images were then synthesized using these light spectra as the spatially averaged spectrum. Specifically, a hyperspectral image *I* was randomly selected from $ds\_dev\_ill\_hal$ and normalized by its mean spectrum. To generate the simulated white reference image $I_s$, the normalized image was element-wise multiplied with the simulated spectrum $f_{\tilde{p}_1,\tilde{p}_2,\tilde{p}_3,\tilde{p}_4}$, thereby preserving the original spatial variations. Concretely, for spatial indices (*i*, *j*), and the channel index *c*, we calculate:

3 $$\begin{aligned} I_s(i,j,c) = f_{\tilde{p}_1,\tilde{p}_2,\tilde{p}_3,\tilde{p}_4}(\lambda _c)\odot I(i,j,c) \oslash \overline{I}(c) \end{aligned}$$

where $\lambda _c$ denotes the wavelength associated to channel *c*, and $\overline{I}$ represents the spatially averaged spectrum of image *I*. To further enhance the coverage of illumination conditions, inter-cluster interpolations based on the acquisitions and simulations were generated, as conducted for LED simulations.

### Neural network training details

As illustrated in Fig. 2, illumination images are multiplied with stray light-free sample images to simulate uncalibrated hyperspectral images under varying lighting conditions. To generate spectrally smooth white reference predictions, a 3D convolutional neural network (CNN) is employed. The network utilizes an autoencoder architecture incorporating ResNet blocks [33] in both the encoder and decoder. Two design choices were critical for the efficacy of the presented method. Firstly, during training, the model is optimized solely on the predicted white reference image, rather than the resulting calibrated sample image, thereby reducing the reliance on the sample dataset. Particularly, the mean-squared error between the predicted and original white reference image serves as the loss function $\mathcal {L}$. Secondly, conventional U-Net-style skip connections between the encoder and decoder are intentionally omitted to minimize the influence of geometric sample information within the predicted illumination image. With these design choices in place, the encoder gradually enlarges the feature dimension from 32 to a 256-dimensional latent space across nine ResNet blocks, using strided convolutions for down-sampling at every third block. The decoder mirrors this process, restoring the original resolution via interpolation. All convolutional layers, except for the input layer, employ a $\text {3}\times \text {3}$ kernel. The model comprises approximately 13.5 million trainable parameters. Optimization was performed using the Adam optimizer with an exponentially decaying learning rate scheduler.

## Experiments and results

We investigated the following research questions (RQs):

1. How do dynamically changing lighting conditions in the OR affect the performance of hyperspectral image analysis?
2. Is the common practice of using spatially uniform lighting models adequate for capturing the complexity of OR illumination in dynamic recalibration?
3. Are neural networks capable of replacing white tile recalibration of hyperspectral cameras in the OR?
4. To what extent can neural network-based recalibration mitigate the performance drop of hyperspectral image analysis under varying lighting conditions?

### Experiment RQ1

**Fig. 4:**
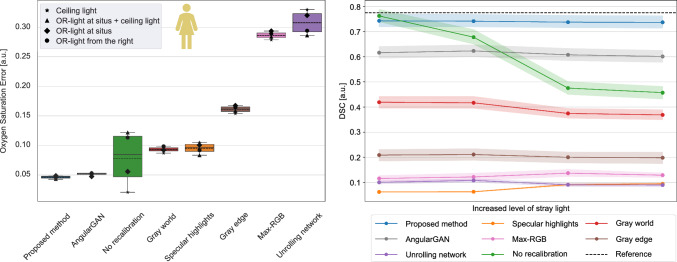
Unlike existing calibration methods, our method produces accurate recalibrations, ensuring robust downstream capabilities on human data. (Left) Absolute errors in organ-specific oxygen saturation estimates between reference images and recalibrated images. (Right) Segmentation results on the recalibrated images. Shaded regions: 95 % confidence intervals

As baseline methods, we used the traditional approaches Gray world [16], Max-RGB [17], first-order Gray edge [18], and an HSI-specific method leveraging specular highlights [19]. Additionally, we incorporated two learning-based calibration techniques: AngularGAN [24], designed for RGB imagery, and an approach [27] tailored for multispectral data, which utilizes unrolling networks to extract surface reflectance. Both learning-based methods were trained on the porcine sample dataset $ds\_dev\_pig$, paired with the acquired illumination images. Semantic organ segmentation and physiological parameter estimation were conducted on in vivo data as downstream evaluation tasks. Stray light-affected versions of the $ds\_test\_pig$ and $ds\_test\_human$ organ datasets were created using the illumination test set $ds\_test\_ill\_hal$, wherein each version corresponded to a distinct illumination configuration of $ds\_test\_ill\_hal$. Subsequently, these images were recalibrated by one of the evaluated methods, followed by inference for the respective downstream task. For segmentation, we employed pig and human organ models sourced from [2, 32], which had been trained on accurately calibrated images. As a segmentation metric, the Dice similarity coefficient (DSC) was used [34] and compared against the performance on the dataset devoid of stray light. In the second downstream analysis, organ-specific physiological parameters, including oxygen saturation, perfusion, hemoglobin, and water index, were calculated according to the procedures outlined in [9]. Reference parameters were obtained by applying the methods to images acquired without stray light. Subsequently, the parameters were computed on the recalibrated images that were originally affected by stray light. Calibration performance was evaluated by the mean absolute error between the recalibrated parameters and their corresponding reference values. For both downstream tasks, the hierarchical structure of the data was respected during aggregation following [1].

The necessity of recalibration is qualitatively demonstrated in Fig. 1 and quantitatively supported by the substantial variance in Fig. 4 when recalibration is omitted. However, existing HSI calibration techniques lacked adequate accuracy. Even the best performing baseline method, AngularGAN, resulted in an average decrease of 21 % in DSC when applied to human data. Similar failures in downstream tasks could be observed on pig and rat images, as demonstrated in Suppl. Figure 3.

### Experiment RQ2

**Fig. 5:**
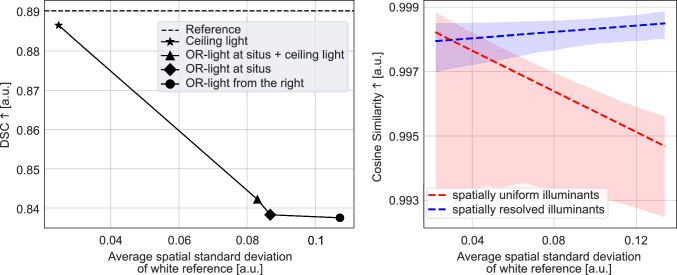
Spatially uniform illuminants cannot adequately represent the surrounding illumination within the OR. Calibration accuracy (right) and downstream performance (left) decrease due to the limitation to spatially uniform illuminants. (Left) Organ segmentation on images that were reilluminated with white reference images and recalibrated with the respective spatially averaged spectrum as spatially uniform illuminant. (Right) Cosine similarities between reference images and images recalibrated using the spatially uniform model (red) and the spatially resolved model (blue). Dashed lines: fitted regression lines; shaded regions: 95 % confidence intervals

To investigate whether spatially uniform illuminants are sufficient for calibration, we first isolated the problem from specific calibration methods. Particularly, images in $ds\_test\_pig$ were relit by multiplying them with white reference images of $ds\_test\_ill\_hal$. Recalibration was then performed using the spatial average of the illumination image as a spatially uniform illuminant estimate. The impact of this reduction was evaluated through organ segmentation, with results compared against reference values obtained without stray light. The left plot of Fig. 5 illustrates the DSC relative to the average spatial standard deviation of the white reference images for the four illumination scenarios in $ds\_test\_ill\_hal$. A clear inverse correlation was observed, with DSC decreasing as the spatial deviation of the corresponding white tile image increased. Crucially, the sole limitation to spatially uniform illuminants resulted in a DSC degradation of more than 9 % in certain lighting conditions. Analogous behavior was observed for oxygen saturation estimation, where the error proportionally increased with the standard deviation (see Suppl. Figure 2).

Furthermore, we performed an ablation study on the illuminant dimensionality within our proposed model architecture by training a comparable model that generates spatially uniform illuminants. For this, our model’s encoder was combined with a pooling operation in the latent space, reducing spatial dimensions, and the decoder was adapted to use 1D convolutional blocks. The training strategy remained similar, except that the illumination images were spatially averaged. For validation, images from $ds\_val\_pig$ were relit by white reference images and subsequently recalibrated using both the spatially uniform model and the proposed spatially resolved model. To gauge calibration performance, the cosine similarity between recalibrated and original images was calculated and illustrated on the right of Fig. 5. Consistent with previous observations, calibration accuracy of the spatially uniform model decreased with increasing spatial standard deviation of the illumination images. In contrast, the spatially resolved model demonstrated stable—if not slightly increased—cosine similarity scores. Although the absolute differences between models appear modest, a statistical t-test confirmed the superior performance of the spatially resolved model ($p=2.57 \cdot 10^{-8}$). Across all ablation studies, cosine similarity scores remained generally high, resulting in only marginal differences between models. However, we observed that even subtle changes in cosine similarity can produce pronounced effects on downstream tasks. Therefore, we recommend using this metric primarily for relative model ranking rather than for interpreting absolute performance values.

### Experiment RQ3

**Table 1:** Our simulation strategy effectively enhances the illumination training data. As validation metric, the spectral cosine similarity between the original and recalibrated images was used. The pig organ validation dataset served as our sample data, augmented with white tile images, which were excluded from downstream analysis. In brackets: 95 % confidence intervals

Illumination Data	Cosine Similarity $\uparrow$
for Training	($\times 100$)
Acquisitions	99.75
	[99.69; 99.80]
+ Simulations	99.79
	[99.76; 99.82]

**Table 2:** Realism-diversity trade-off for simulated illuminations. The sampling range of simulation parameters is varied. $n_{\sigma }=0.25$ yields the highest cosine similarity, indicating an optimal balance. In brackets: 95 % confidence intervals

Standard Deviations of	Cosine Similarity $\uparrow$
Parameter Range	($\times 100$)
$n_{\sigma }=0.1$	99.78
	[99.75; 99.80]
$n_{\sigma }=0.25$	99.79
	[99.76; 99.82]
$n_{\sigma }=1$	99.69
	[99.62; 99.76]

**Fig. 6:**
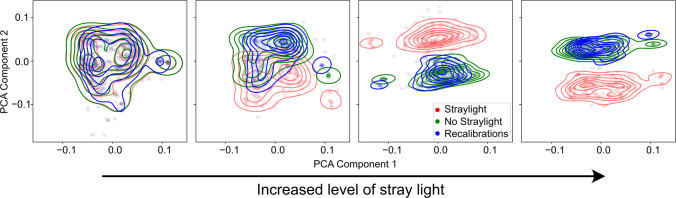
The proposed model produces recalibrations (blue) from stray light-affected images (red) that resemble corresponding non-stray light-affected images (green) within the latent space. The features were extracted using the model’s encoder, pooled into 1D feature vectors, and then subjected to PCA

The model’s illumination calibration capabilities were evaluated using a downstream-invariant approach across in-distribution and out-of-distribution settings. In-distribution performance was examined by analyzing latent features generated by our model. To achieve this, illumination conditions of $ds\_test\_ill\_hal$ were simulated within the images of $ds\_test\_pig$. Subsequently, recalibration was performed using our trained model. Finally, the original, stray light-affected, and recalibrated images were encoded into the latent space via our trained encoder, and the first two PCA components were visualized using kernel density estimation. This visualization was conducted for each stray light scenario, as illustrated in Fig. 6. The model’s ability to differentiate between the original and stray light-affected images correlated with the level of stray light within the illumination images, suggesting meaningful feature extraction. Furthermore, the distribution of our recalibrations closely resembled the original distribution, indicating high calibration accuracy.

To further assess the calibration accuracy in an out-of-distribution setting with a highly reliable reference, we recalibrated the colorchecker board dataset $ds\_test\_cc$ using our model. As illustrated in Suppl. Figure 2, the proposed method achieved the highest average cosine similarity (0.9905) among the baseline methods from Section 3.1, closely approaching the gold standard white tile calibration (0.9945).

The model’s design choices, particularly the impact of halogen-based illumination simulations, were analyzed via an ablation study on the validation set $ds\_val\_pig$. Table 1 shows that incorporating simulations to real data improved performance, demonstrating the effectiveness of our simulation strategy. This benefit also translated to downstream applications, where segmentation performance increased by $+0.05$ DSC on the validation set. To determine the hyperparameter $n_\sigma$ that controls widths of the parameter ranges, we tested three values. As can be seen in Table 2, large values of $n_\sigma$ led to unrealistic simulations and performance drops. Finally, the architectural choice of omitting skip connections is analyzed in Suppl. Table 1.

### Experiment RQ4

**Fig. 7:**
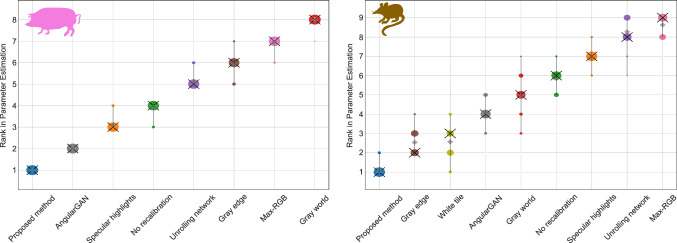
Our model showcases the highest robustness against simulated and real stray light interference in the estimation of physiological parameters. The accompanying plot evaluates calibration models on pig (left) and rat (right) organ images by ranking them according to the absolute error of four physiological parameters. Each blob’s area reflects the relative frequency at which the rank is achieved across 1000 bootstrap samples, following [35]

To assess the downstream capability of our method, we replicated the experiments outlined in Section 3.1, incorporating our recalibration approach. Following the findings presented in Table 2, the hyperparameter $n_\sigma$ was set to 0.25 for all experiments, which were carried out on untouched sample and illumination test sets. As shown in Figs. 4 and 7, and in Suppl. Figure 3, our method outperformed previous methods by a large margin. In particular, the proposed approach yielded high segmentation scores on pig and human organ images across diverse illumination setups. Furthermore, the model excelled in the parameter estimation task, consistently ranking first on pig and rat datasets compared to the baseline methods. This was further supported by the lowest average absolute error in oxygen saturation, shown in Fig. 4, and the stable hemoglobin estimates across real stray light scenarios, illustrated in Fig. 1.

## Discussion

Our study is the first to provide in vivo evidence that dynamic illumination changes in the OR can lead to severe failures in HSI downstream analyses. The clinical implications are substantial, as manual recalibration considerably impedes the efficiency of surgical workflows and may limit the widespread adoption of HSI cameras in clinical practice. Notably, the proposed method stands as the only calibration method in our study that consistently achieves high accuracy regardless of the downstream task and domain, thereby highlighting its high clinical applicability. It further offers several key conceptual benefits. First, white reference measurements are not only impractical due to sterilization and workflow constraints, but they are also susceptible to oversaturation, which accounts for the suboptimal performance observed on the rat data (see Fig. 7). In contrast to the majority of existing methods, our approach accommodates spatial deviations in illumination, thereby enabling enhanced calibration accuracy (see Fig. 5). A key strength of the presented approach is its inherent generalizability. It exhibits superior performance to the competing neural network method, AngularGAN, even under identical training conditions—a performance gap we attribute to the intrinsic domain shift in auto-encoded images. Consequently, our model’s ability to seamlessly handle human data without sacrificing calibration accuracy reinforces its applicability in clinical settings (see Fig. 4). Additionally, our algorithm is well-suited for practical deployment, processing each image in approximately 0.71 s of which only 0.06 s is required for model inference on a NVIDIA TITAN RTX GPU. With a modest GPU memory footprint of about 1.4 GB, integration into existing surgical HSI systems is both feasible and efficient.

While our work may be limited by not addressing every conceivable illumination scenario, we prioritized key light sources prevalent in ORs and validated our model on highly diverse datasets. Consequently, we are confident that our conclusions will hold in a wide range of settings.

In conclusion, this study introduces a novel learning-based illumination calibration method for spectral imaging. Our methodology not only delivers superior performance compared to existing approaches across diverse settings but also lends itself to seamless integration into HSI systems for ORs. This advancement could thus facilitate the emergence of surgical workflow-optimized spectral imaging.

## Supplementary Information

Below is the link to the electronic supplementary material.

Supplementary file 1 (pdf 725 KB)
