# Supplementary material for: Neural illumination calibration for surgical workflow-optimized spectral imaging
Source: Int J Comput Assist Radiol Surg. 2025 Oct 7;21(4):665–75. doi: 10.1007/s11548-025-03525-8 (PMC13194271; doi:10.1007/s11548-025-03525-8)
Supplement: Supplementary file 1 — (pdf 725 KB) [file 11548_2025_3525_MOESM1_ESM.pdf]

Supplementary Material

Neural Illumination Calibration for Surgical Workflow-Optimized Spectral Imaging

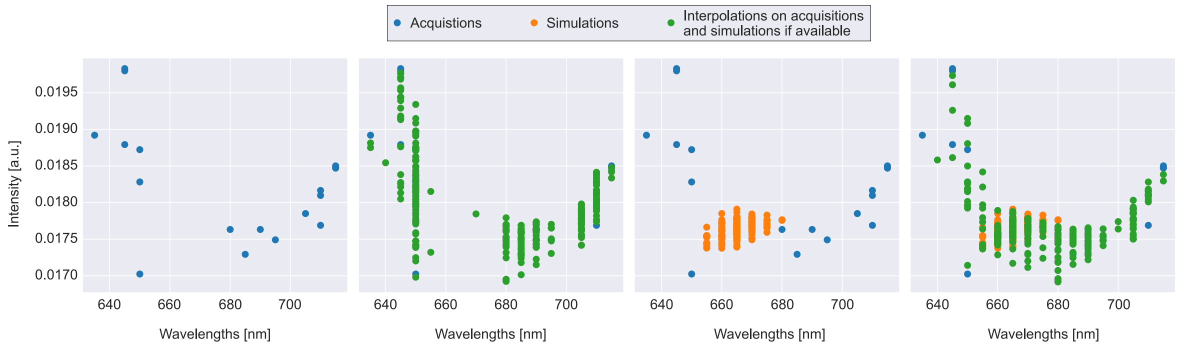

Figure 1: Our simulation strategy increases variability in the positions of spectral extrema, whereas interpolation alone only alters their values. The plots show maxima positions of illumination spectra (x-axis) against their normalized values (y-axis). Acquired measurements alone provide sparse coverage of possible combinations (1). Interpolating from acquisitions (2) modifies only the maxima values while keeping their positions fixed. In contrast, our physics-based simulations introduce additional variability in maxima positions (3). Combining acquisitions with simulations and subsequent interpolation (4) yields the most comprehensive coverage of both maxima positions and values.

Model variant	Cosine Similarity (×100)
With encoder-decoder skip connections	99.73 [99.70; 99.75]
Without encoder-decoder skip connections	99.79 [99.76; 99.82]

Table 1: Removing U-Net skip connections improves recalibration. Spectral cosine similarity between original and recalibrated images was used as validation metric. Results are shown on the pig organ dataset with additional white tile images (excluded from downstream analysis). In gray: 95 % confidence interval.

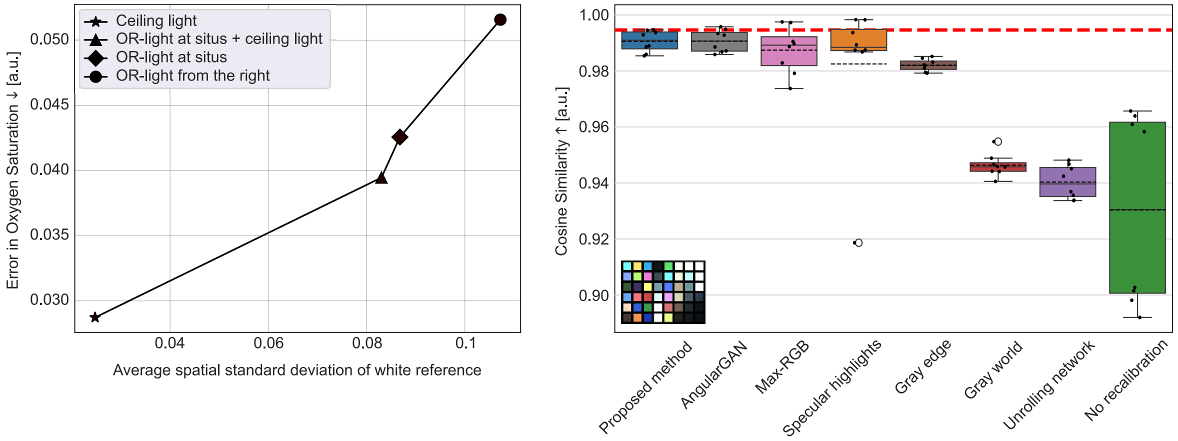

Figure 2: High calibration accuracy, demonstrated by the proposed model on phantom data, necessitates spatially resolved illuminants. (Left) Oxygen saturation estimation on porcine organ images that were relit with white reference images and recalibrated with the respective spatially averaged spectrum. (Right) Cosine similarities between reference and recalibrated color spectra (aggregated over colors) indicate superior performance with our spatially resolved model. Red dashed line: White tile calibration.

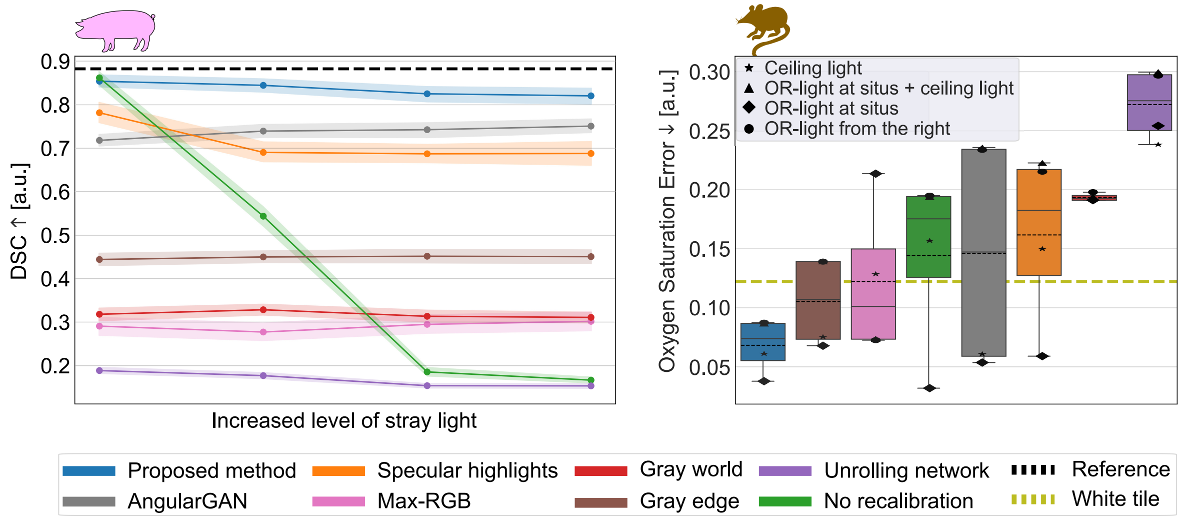

Figure 3: Our model showcases superior robustness against both simulated and real stray light interference. (Left) Organ Segmentation on recalibrated porcine organ images subjected to simulated stray light. Shaded regions: 95 % confidence intervals. (Right) Absolute oxygen saturation errors between reference rat organ images and corresponding recalibrated images contaminated with real stray light.
